# A Case Report on Management of Liver Cirrhosis Using Ayurveda and Integrative Approach of Treatment

**DOI:** 10.1155/crhe/1176751

**Published:** 2024-11-27

**Authors:** Prasan Shankar, Bhavya Vijay, Bhargavi, Mahima Rahman, Kimi Anand, Vasudevan Nampoothiri, Prashanth

**Affiliations:** ^1^Department of Rasayana Tantra, I-AIM Healthcare Center, The University of Trans-Disciplinary Health Sciences and Technology, Bangalore, Karnataka, India; ^2^Center for Community Health, Clinical Research, and Education, I-AIM Healthcare Center, The University of Trans-Disciplinary Health Sciences and Technology, Bangalore, Karnataka, India

**Keywords:** ascites, Ayurveda, buttermilk, integrative care, liver cirrhosis, Panchakarma, Udara

## Abstract

**Background:** Liver cirrhosis is an advanced stage of abnormal fibrogenesis of tissues that causes liver injuries. Though cirrhosis can be managed by etiological parameters, its long-term reversal is still a question. Ayurveda system of medicine diagnoses liver disease under “Kamala” and “Udara” with promising outcomes of treatment. This case series discusses three cases of liver cirrhosis where internal Ayurvedic medications and external therapies including “Panchakarma” (a treatment approach of detoxifying and rejuvenating) resulted in effective management of the disease.

**Case Presentation:** Three cases of decompensated liver cirrhosis were treated at an Ayurveda hospital. Relevant examinations and investigations were done, and patients were monitored at regular intervals. Patients were treated with Ayurvedic therapies and were monitored for changes using standardized tools of assessment.

**Conclusion:** In all three patients, there was an improvement in quality of life and a reduction in symptoms such as abdominal pain, transpyloric diameter, pedal oedema, and fatigue, as well as a significant reduction in liver function test parameters. Decompensated liver cirrhosis can be managed with an Ayurvedic treatment regimen that includes Ayurvedic medications, Panchakarma, along with a proper diet regimen with salt and fluid restrictions. This case series concludes that while cirrhosis is not completely reversible, fibrosis could be reversed. The support of modern medicine for monitoring and emergency care remains paramount. Furthermore, proper documentation of all the observations can help in assessing the outcomes of Ayurveda therapies and aid in developing integrative protocols for the management of liver cirrhosis in the future.

## 1. Introduction

Cirrhosis, an advanced stage of liver fibrosis, is the development of regenerative nodules due to abnormal fibrogenesis caused by liver injuries [1]. It is a chronic liver disease with high morbidity and mortality rates. The Global Burden of Disease (GBD) in 2017 reported an average of 2.4% of deaths due to liver cirrhosis [2]. The two major etiological factors of liver cirrhosis in India and other Asian countries are alcoholic liver disease and hepatitis [3, 4]. Cirrhosis causes increased fibrogenesis which shunts the blood supply, resulting in vasoconstriction, hypertension, and salt retention, and eventually progresses toward liver failure and death [1]. The treatment of liver cirrhosis often involves long-term etiological management of symptoms and liver transplantation at the end-stage of liver damage.

According to the Ayurveda Classification of Disease (ACD), liver cirrhosis is categorized under *Udara.* Various published articles report the success in the effective management of liver diseases [5–9]. In the present case series, three cases of liver cirrhosis successfully managed with Ayurveda treatment are discussed.

## 2. Case Presentations

All patients were treated at an Ayurvedic Healthcare Center. Patient's treatment and investigation data were extracted for this study. To protect patients' privacy, all patient-related information has been deidentified. Written and informed consent has been taken from the patients or their caretakers for publishing these data. The Institutional Review Board of the Institute of Ayurveda Healthcare Center approved this retrospective case series.

### 2.1. Case 1

#### 2.1.1. Case Presentation

A 43-year-male patient presented with yellowish discoloration of the sclera and urine, distension of the abdomen, reduced appetite, and pain in the abdomen for over a month and a half. The complaints were associated with swelling in the lower limbs for 4 days. He had been a chronic alcoholic for 15 years and had been consuming about 90 mL of alcohol daily over the last 6 months. In addition, the patient has reported tobacco use for 3 years.

#### 2.1.2. Diagnosis and Treatment History

An ultrasound scan revealed early cirrhosis of the liver along with gall bladder calculus. He was admitted for standard medical care during which he started to develop withdrawal symptoms of alcohol. He also took herbal medications from a folklore practitioner during his treatment. However, the patient was not responding to the interventions; hence, he approached the Ayurveda Healthcare Center for further help.

#### 2.1.3. Diagnostic Assessments

**Figure 1:**
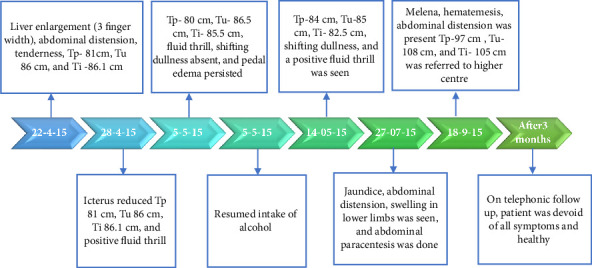
Case 1 timelines of treatment. Tp = transpyloric diameter, Tu = transumbilical diameter, Ti = transiliac diameter.

At the healthcare center, based on the clinical presentations, examinations, and investigations, the patient was diagnosed with decompensated alcoholic liver cirrhosis under allopathic diagnostic criteria. Based on the ACD, he was diagnosed with *Kamala* with *Udara*. He presented to the hospital with severe abdominal distension, tenderness over the right hypochondriac region, liver enlargement (up to three fingers), dullness on percussion, shifting dullness, eversion of umbilicus, and fluid thrill. Further laboratory investigations indicated liver cirrhosis (Figure 1). MRI of the abdomen revealed alcoholic hepatitis.

#### 2.1.4. Therapeutic Interventions

**Figure 2:**
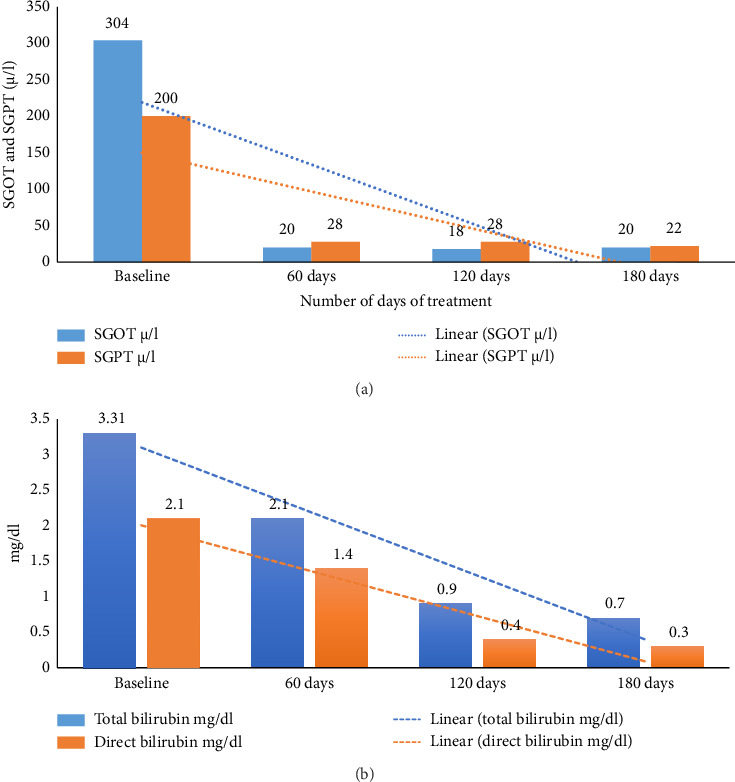
(a) Timeline of changes in SGOT and SGPT values. (b) Timeline of changes in total and direct bilirubin values.

On initiation of treatment, the patient was administered a disease-specific diet along with internal medications (Annexure 1: Supporting Table 1). Within 7 days, the patient reported an improvement in appetite. Progressively, other symptoms such as yellowish discoloration of sclera and abdominal distention subsided. Within 45 days of treatment, fluid thrill was absent, he had a reduction in transpyloric, transumbilical, and transiliac diameter (Table 1). Liver function test parameters also showed a significant reduction over time (Figures 2(a) and 2(b)). The patient exhibited quick recovery during the treatment and was discharged after 45 days of intervention. The patient was only under Ayurvedic medicine during his treatment, no supplementary biomedicine was administered during this time (refer Annexure 1: Supporting Table 1 for treatment chart).

#### 2.1.5. Follow-Up and Outcomes

Within 2 months of discharge, the patient once again presented with abdominal distension and yellowish discoloration of the sclera with a positive fluid thrill. He had resumed intake of alcohol. Due to the relapse of symptoms, abdominal paracentesis was done. During the treatment, the patient developed fever; he was symptomatically treated for the same. Later, as he developed signs of melena and hemoptysis, he was referred to a higher center for further management. Telephonic follow-up revealed that banding was performed along with paracentesis. Thereafter, the patient was given Ayurveda oral medications for 3 months. Patient recovered fully thereafter and is currently still alive and healthy. Timelines of treatment have been mentioned in Figure 1.

### 2.2. Case 2

#### 2.2.1. Case Presentation

An 80-year-female presented with symptoms of puffiness in face, body swelling, endometrial thickening, and vaginal bleeding. The patient was overweight, with no history of diabetes mellitus or hypertension.

#### 2.2.2. Diagnosis and Treatment History

The patient was apparently healthy 1 year before the onset of symptoms. Initially, the patient had vomiting and loose stools, with fever presenting on and off, and had consulted with a local biomedical practitioner for treatment of the above symptoms. The patient was overweight, with no history of diabetic mellitus or hypertension.

#### 2.2.3. Diagnostic Assessments

The laboratory investigations indicated an increase in SGOT and SGPT to 766 *μ*/L and 35 *μ*/L, respectively. Alkaline phosphatase was 215 *μ*/L with TSH of 5900 IU/mL. Furthermore, an ultrasound of the abdomen and pelvis confirmed chronic liver disease with portal hypertension and ascites, gallbladder thickening, and splenomegaly.

#### 2.2.4. Therapeutic Interventions

**Figure 3:**
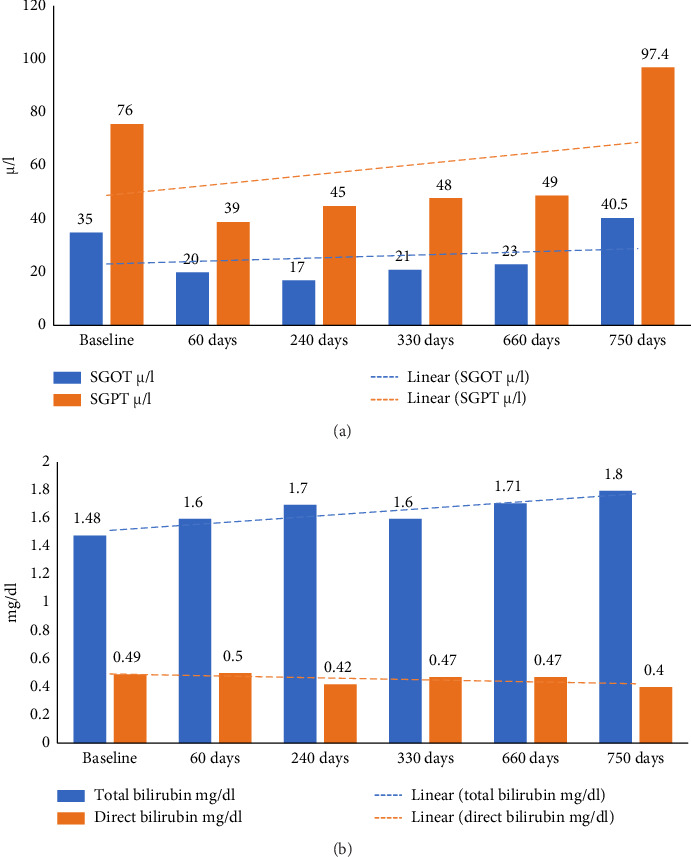
(a) Timeline of changes in SGOT and SGPT values. (b) Timeline of changes in total and direct bilirubin values.

As per ACD, she was diagnosed with *Udara*. The patient was primarily treated using internal medications and milk- and buttermilk-based diet (see Annexure 1: Supporting Table 2). By the end of 60 days of treatment, swelling and breathlessness had reduced, appetite had increased, and fatigue and tiredness had reduced. SGOT, SGPT, and alkaline phosphatase also reduced significantly (Figures 3(a) and 3(b)). Abdominal USG scan also showed liver size to be normal (13.1 cm) with the absence of ascites. The patient was treated on an outpatient basis, and at the end of treatment, the patient was advised to continue the same medications and strictly adhere to the milk and buttermilk diet. The patient was only under Ayurvedic medicine without any supplementary biomedicine during this time (refer Annexure 1: Supporting Table 2 for treatment chart).

#### 2.2.5. Follow-Up and Outcomes

**Figure 4:**
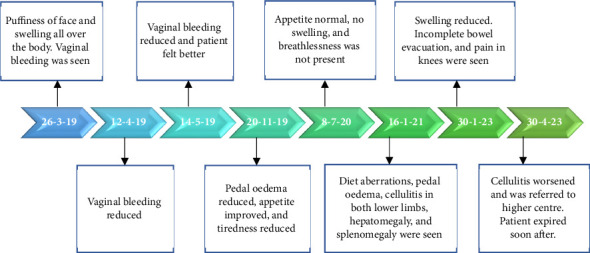
Case 2 timelines of treatment.

During follow-up at the end of 8 months, it showed that pedal oedema had reduced completely. From the time of initiation of treatment until about 1 year 2 months, the patient's health status continued to improve. Thereafter the patient delayed her follow-up for about 5 months and was not regular with medications and diet as reported by her son. Due to diet aberrations, she once again presented with pedal oedema. In addition, she developed a new complaint of cellulitis on both lower limbs. Ultrasound scan reported hepatomegaly and splenomegaly. She was once again advised to take the same medications along with milk and buttermilk diet. Her health improved within 15 days with a reversal in oedema, and she remained healthy for over a month. Telephonically, it was informed that due to the progression in severity of cellulitis, she was referred to a higher cente rfor management. The patient's health continued to worsen and soon after that the patient expired (Figure 4).

### 2.3. Case 3

#### 2.3.1. Case Presentation

A 54-year-male patient came with complaints of abdominal pain with swelling in both lower limbs. Physical examination revealed distension of the abdomen, fluid thrill, everted umbilicus, and an abdominal girth of 99 cm.

#### 2.3.2. Diagnosis and Treatment History

The patient had been a chronic alcoholic for 20 years (Brandy—150 mL daily). Abdominal ultrasonography reported shrunken liver (9.8 cm) and enlarged spleen (13.1 cm), confirming alcoholic liver cirrhosis, splenomegaly, and ascites. The patient comes from a lower income family.

#### 2.3.3. Therapeutic Intervention

The patient presented with swelling of the abdomen, pedal oedema, and was diagnosed as Kamala with Udara as per ACD. During his visit to the Ayurveda Healthcare Center, considering that the patient had a distended abdomen, he was referred to a higher center for further evaluation. He was advised paracentesis, immediately after which he started Ayurveda intervention.

**Figure 5:**
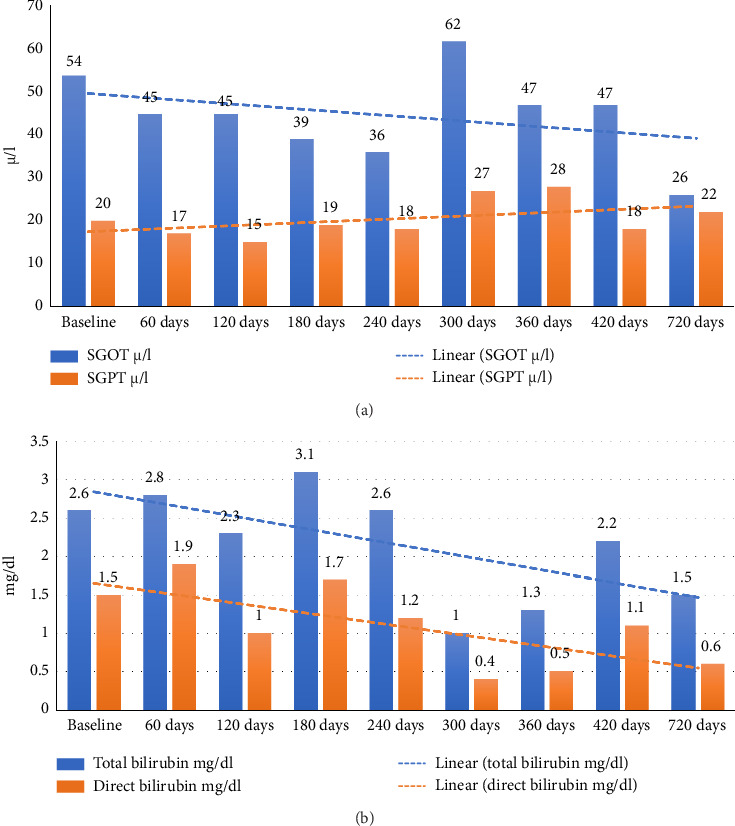
(a) Timeline of changes in SGOT and SGPT values. (b) Timeline of changes in total and direct bilirubin values.

The patient was treated with oral interventions, Panchakarma, and external treatments. He was admitted to the healthcare center where after a prolonged inpatient stay and intensive Ayurveda treatments spanning over 8 months, the patient's abdominal distension, pedal oedema, and fluid thrill gradually showed signs of resolution with improvement in liver function test parameters as well (Figures 5(a) and 5(b)). Abdominal girth was reduced from 99 cm to 78 cm and appetite improved. Patients' treatment and diet were altered at different time points during the treatment based on the changes observed. In the ninth month of management, the patient had bleeding per rectum, abdominal distention, and swelling in the legs. He was referred to a higher center with suspicion and treatment of melena. The patient was only under Ayurvedic medicine during his treatment (refer Annexure 1: Supporting Table 3 for treatment chart).

#### 2.3.4. Follow-Up and Outcomes

**Figure 6:**
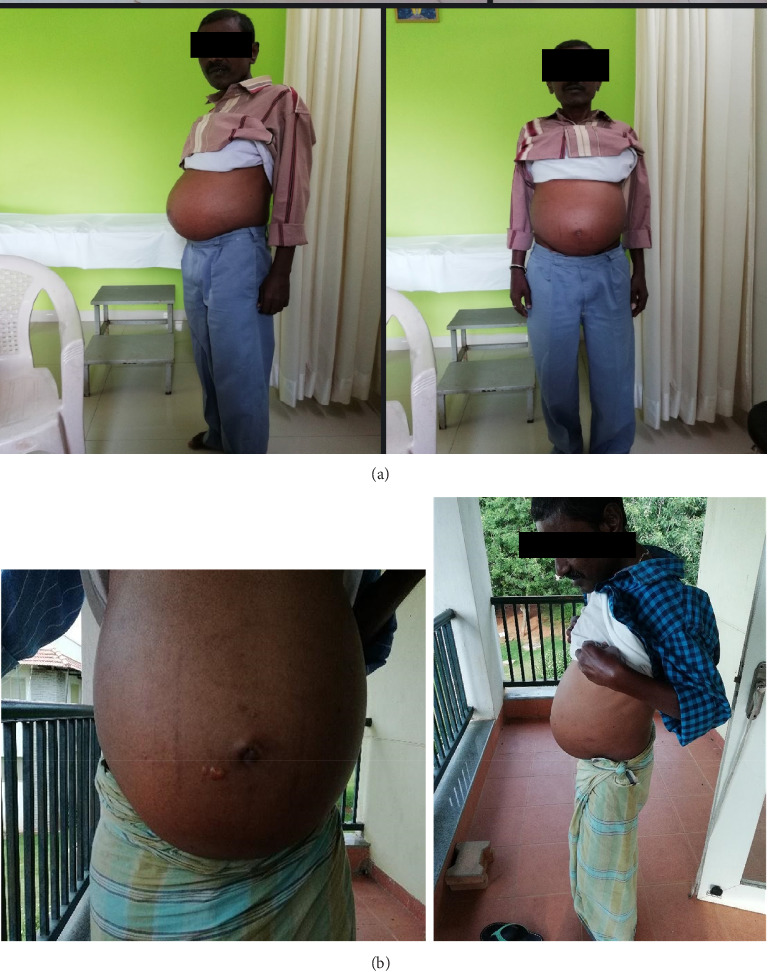
(a) Before treatment and (b) after 2 months of treatment.

**Figure 7:**
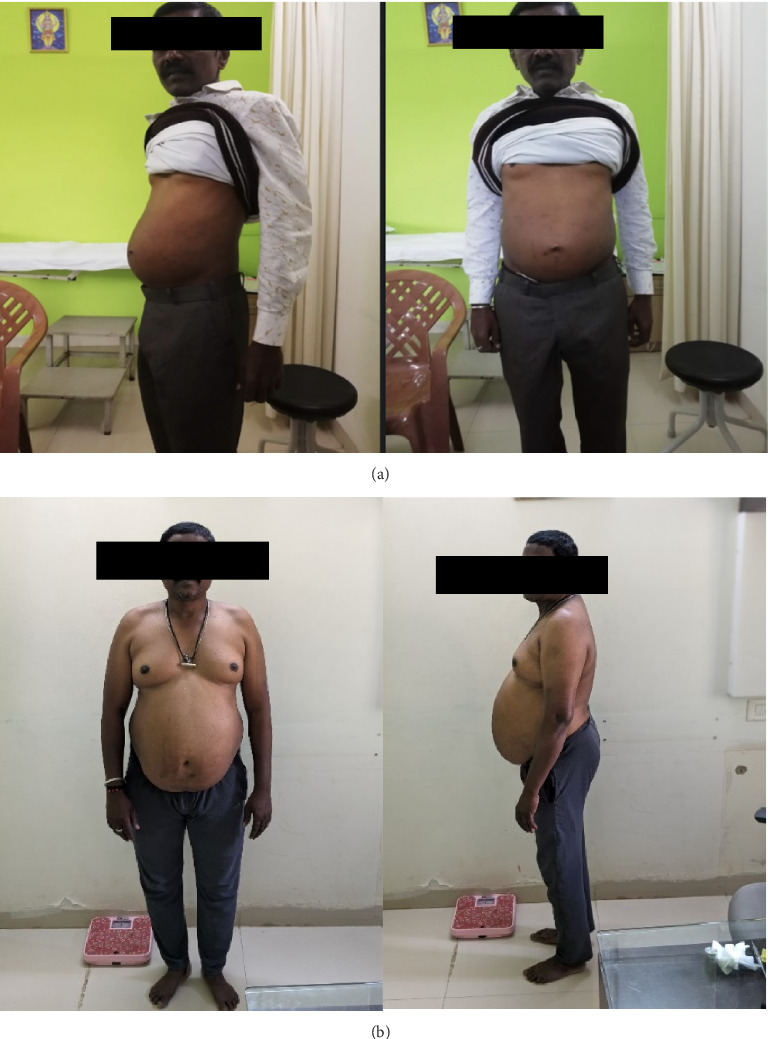
(a) Patient after 4 months of treatment and (b) patient after 2 years of treatment.

After melena had resolved, he was once again admitted to the Ayurveda Health Center for inpatient treatment. Abdominal ultrasonography showed an altered liver size of 11.8 cm; however, spleen enlargement still persisted (16.3 cm). As of today, all of the clinical findings have been reversed and is presently healthy (Figures 6 and 7).

### 2.4. Adverse Events

**Figure 8:**
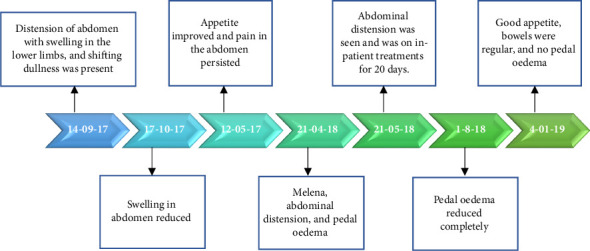
Case 3 timelines of treatment.

During the course of treatment, the patient presented with melena. The patient was immediately referred to his biomedical practitioner for further management. Once his condition was stable, the patient resumed Ayurveda medication (Figure 8).

## 3. Discussion

Approximately 60% of cirrhotic patients develop ascites within 10 years of diagnosis [10]. Further complications such as jaundice, portal hypertension, and gastroesophageal varices, if left untreated often progress to portopulmonary hypertension, hepatopulmonary syndrome, hepatorenal syndrome, hypernatremia, coagulation abnormalities, venous thromboembolism, and hepatic encephalopathy. It has been observed that the one-year survival rate is 56% in patients with ascites. A total of 1%–5% of these patients can progress to hepatocellular carcinoma (HCC) [11]. As a reflection of the high mortality associated with decompensated cirrhosis, much of the outpatient management of these patients is focused on the prevention of these complications. Thus, a rational application of multidimensional integrative treatments could aid in optimal treatment in challenging conditions such as liver cirrhosis, as presented in the case series. Cirrhosis is a response to chronic liver injury which involves development of regenerative nodules surrounded by fibrous bands. Major clinical consequences are impaired hepatocyte function, portal hypertension, and HCC. Hepatitis B, Hepatitis C, and alcohol abuse are the most common causes of liver cirrhosis among others. Environmental and host factors have an important role in the prognosis of liver cirrhosis [1]. Asymptomatic liver disease (compensated liver disease) when treated early has a better chance of reversal. In comparison, overall mortality in symptomatic liver disease (decompensated liver disease) is as high as 85% over 5 years without transplantation. Studies have showed a reversal of fibrosis, but complete regression of cirrhosis has not been clearly documented [12].

### 3.1. Ayurveda Rationale

**Figure 9:**
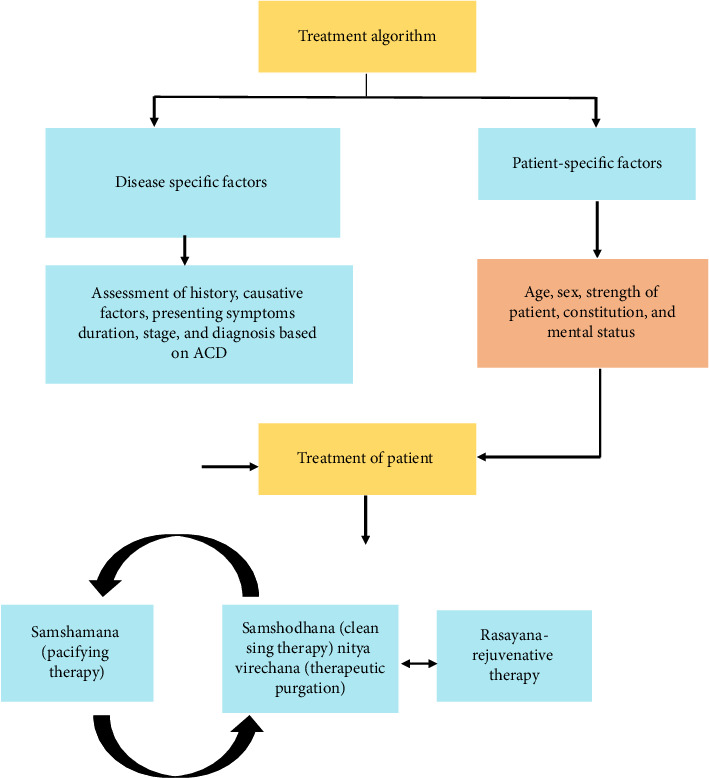
Treatment algorithm.

The liver is a peculiar organ that can regenerate itself after damage. The liver is known as *Yakrit* in Ayurveda and is the seat of pitta dosha. An excess intake of factors aggravating pitta causes inflammation and toxicity in the liver. The algorithm of treatment is shown in Figure 9. The treatment approaches aim at pacifying pitta dosha where *Snehapāna*, *Virechana*, followed by *Rasāyana* are administered to rejuvenate the liver [8]. Historically, Ayurveda has demonstrated its relevance in the treatment of liver diseases in both institutional and noninstitutional settings. For instance, Nobel laureate Baruch Blumberg's work on *Phyllanthus niruri* was inspired by local health practices [5].

Cardinal symptoms of Udara (ascites) described in the texts of Ayurveda are (Sushka Vaktra) dryness of the mouth, (Krishe Krisha gatra) emaciated extremities, (Adhamata udara Kukshayah) distended upper and lower abdomen, (pranashta agni) severe loss of appetite, (pranashta bala) severe loss of strength, (pranashta ahara) severe loss of nourishment, (sarva chetasu deenah) apathy and limitation of physical activities, and (Jala purna driti parsha) fluid thrill (dull on percussion). The treatment was administered based on the principles of Udara chikitsa as described in Charaka Samhita [13].

The primary goal of intervention in all three cases was to eliminate fluid accumulation (Udaka and Kapha) and restore tissue health (Dhatu Samya), improve digestion and metabolism (Deepaniya), ensure proper nutrition, and ultimately restore doshic balance. To achieve this, an ingenious strategy of frequent purgation (Nitya Virechana), diuretics (Mutrala) using various formulations, has been advised as a base treatment regimen in all three cases. Mutravirechaneeya dravyas such as Gokshura (*Tribulus terrestris Linn.*), Gomutra (cow's urine), Ksheera (Milk), and Ikshu *(Saccharum officinarum Linn)* are said to cause virechana of mutra or diuresis due to active principles present in them. Gokshura contains potassium alkali, which is found to cause diuresis. Similarly, Arjuna (Terminalia arjuna Roxb) contains a triterpinoid saponin called arjunlic acid and Punarnava (*Boerhavia diffusa* Linn.) contains Punernavoside which produces diuresis [14]. This principle has shown effective results in all three cases of liver cirrhosis in this study. In Case 1, the change in abdominal girth is observed in 45 days of treatment (Table 1). In Case 2, improvements in health with respect to symptoms such as puffiness of the face, vaginal bleeding, pedal oedema, tiredness, and breathlessness were observed. Whereas in Case 3, reduced ascites, pedal oedema, and abdominal girth as evidenced by the patient's treatment timeline. Symptomatic relief is observed in all three cases.

Table 2 summarizes the list of common herbs and their application in the treatment of all three cases of liver cirrhosis. Over the last several decades, there have been many preclinical studies on hepatoprotective herbs used clinically [5, 8, 26–31]. Piperine, an active ingredient of *Piper longum*, attenuated acetaminophen-induced hepatotoxicity. *Piper longum* (Pippali) was examined for its hepatoprotective effects and found to offer significant protection to the liver by preventing the leakage of intracellular enzymes by its membrane stabilizing and antioxidant activity recorded by ASL, ALT, ALP, and lipid peroxidase values [32]. *Piper Longum* is often administered in increasing and tapering doses referred to as Pippali Rasayana [13] Similarly, *Picrorhiza kurroa* (Katuki) has demonstrated hepatoprotective and anti-inflammatory effects [33]. Punarnavastak kwath, an Ayurvedic formulation made of drugs such as Punarnava (*Boerhavia diffusa*), Neem (*Azadirachta indica*), Patola (*Trichosanthes dioica*), Shunthi (*Zingiber officinale*), Kalmegh (*Andrographis paniculata*), Giloy (*Tinospora cordifolia*), Devdaru (*Cedrus deodara*), Haldi (*Curcuma longa*), and Haritaki (*Terminalia chebula*) has exhibited anti-inflammatory and hepatoprotective effects [21] in CCl4-induced hepatotoxicity by decreasing levels of serum liver marker enzymes such as aspartate transaminase, serum alanine transaminase, serum alkaline phosphatase, and serum bilirubin and an increase in the protein level due to the rich source of antioxidants [34]. *Eclipta alba* (Bhringaraja) has also been widely studied in liver damage [35]. Though preclinical research has validated the immunomodulatory, hepatoprotective activities of several herbs, clinical research on the subject is limited.

In addition to this, a special diet consisting of buttermilk and/or milk is commonly advocated as part of the diet to modulate gut health (Agni) and restore homeostasis. Takram or buttermilk has a special place in the management of Udara. It is useful and prescribed widely for ascites, and pedal oedema, swelling and fluid accumulations, and obesity are amongst many other indications of buttermilk [13].

Buttermilk (*Takra*) by virtue of its properties (*gunas*), which match the above algorithm, is considered an elixir (amrita) for patients with ascites (Udari). Rice, oats, ragi, and rava which are easily digested (laghu) are administered with buttermilk as the main medium of delivery.

One of the key patient specific factors that helps patients choose between milk or buttermilk is strength (Bala). A person with good strength (Bala) would be an obvious choice for a buttermilk diet. On the other hand, a person who is weak (bala heena) would be advised to drink milk.

Green gram (*Vigna radiata*), red lentil (*Lens culinaris*), pigeon peas (*Cajanus cajan*), and soup of lean meats have also been advised. The importance of this diet today is also endorsed by nutritionists, as pulses/lean meats are an important source of proteins. Possibly, the choice of protein and “food form and design” advocated also ensure there is no disruption of digestion and metabolism.

External anointments over the abdomen and abdominal strapping have also been advised in order to prevent vata aggravation. Thus, internal medications, diet, and external methods are employed to achieve this goal.

Among the cases described, one patient died, and two patients, despite various complications, the condition improved with respect to overall quality of life and continued to survive till date.

### 3.2. Limitations of the Study

A case series has the advantage of describing the complete diagnostic and treatment approaches in enormous detail; however, this study is limited by the fact that it is a small sample. While Ayurveda was primarily used in the treatment, some integrative treatment methods were incorporated for emergency medicine (paracentesis, banding, and use of essential internal medications). Along with Ayurveda diagnosis and observations, standard biochemical blood tests and symptomatic interventions were incorporated. Furthermore, complications such as hemoptysis and melena, observed in some cases, are known to occur in cirrhotic patients and need modern diagnostic and technological interventions to be treated efficiently.

It was therefore clear that an integrative approach was better suited to manage the case comprehensively. In these patients' journeys toward recovery, the role of both systems was crucial.

## 4. Conclusion

This case series shows the challenges in cirrhosis treatment and why it is not completely reversible, a dual between dogma and myth. Decompensated liver cirrhosis can be managed effectively with an Ayurvedic treatment regimen that includes Ayurvedic medications, Panchakarma, and a proper diet regimen with salt and fluid restrictions. The support of modern medicine for monitoring and emergency care remains paramount.

Furthermore, proper documentation of all the observations, appropriate investigations at proper intervals, and interpretation of results can demonstrate the effectiveness (reversal of fibrosis/cirrhosis) of the Ayurveda treatments and help develop integrative protocols for the management of liver cirrhosis in the future.

The future prospects from this study are to conduct larger retrospective studies or randomized clinical trials assessing the efficacy of Ayurveda and integrative treatments using control and test groups receiving standard of care to establish effectiveness of the treatment methods. Both in vitro and in vivo studies can be carried out to understand the mechanisms of action of the treatment algorithms and approaches used in Ayurveda.

### 4.1. Patient Perspective

• Case 1: Patient recovered fully and is currently still alive and healthy. The patient visited the health center a few years after recovery to express his gratitude to the doctors.
• Case 2: Patient's son expressed his happiness with Ayurveda treatment and reported that her health was at its best under Ayurvedic care.
• Case 3: The patient reported that he used to have reduced appetite, distended abdomen, swelling in legs, pain in the abdomen, fatigue, and used to feel tired after walking for a short distance. Conventional medications alone did not help him. An integrative approach is what helped him. After taking ayurvedic medications, all the above symptoms reduced, he gained strength, swelling in legs and pain reduced, and is continuing doing fine. He also affirmed that he is following a strict diet and has stopped alcohol intake.

## Figures and Tables

**Table 1:** Pre–post assessment of abdominal girth.

	Before (in cm)	After 45 days of treatment (in cm)
Transpyloric diameter	80	80
Transumbilical diameter	90	86.5
Transiliac diameter	90.5	85.5

**Table 2:** Herbs used in the treatment and their pharmacological principle of action.

Sl. no	Botanical name	Pharmacological activity according to Ayurveda	Biological activity
1	*Adhatoda vasica*	Rakta-pitta śamanam (rakta-pitta pacifying) and kapha-śamanam (kapha-pacifying)	Hepatoprotective and decreased levels of SGOT and SGPT [15]

2	*Boerhavia diffusa*	Rakta-pitta śamanam and dīpanam (appetizing action)	Hepatoprotective, reduction in SGOT, SGPT, ACP, and ALP levels [16]

3	*Tinospora cordifolia*	Rasāyana (rejuvenation therapy), vata-pitta śamanam(vata-pitta pacifying), and dīpanam (appetizing action)	Prevention of fibrotic tissue deposition and activation of Kupffer cells [17]

4	*Phyllanthus amarus*	Rakta-pitta śamanam(kapha-pitta pacifying)	Increases MMPs (matrix metalloproteinases) activity in hepatic fibrosis [18]

5	*Piper longum*	Dīpanam, rasayana, and vata-kapha śamanam (vata-kapha pacifying)	Hepatoprotective [19], antifibrotic [3], and anti-inflammatory [3] activities.

6	Triphala *Terminalia chebula* *Terminalia bellirica* *Emblica officinalis*	Kapha-pitta śamanam and dīpanam	Hepatoprotective, free radical scavenging, reduction in overexpression of TNF-*α*, IL-6, and anti-inflammatory activity [20]

7	*Picrorhiza kurroa*	Dīpanam and kapha-pitta śamanam	Decreasing lipid content of the liver tissue, morphological regression of fatty infiltration, hypolipidemic activity, and reduction of cholestasis [21]

8	*Phyllanthus emblica*	Tridoṣaḥ śamanam (tridosha–pacifying), rasāyana, and rakta-pitta śamanam	Hepatoprotective, lowered lipid peroxidation, nitric oxide levels, and elevated antioxidant status [22]

9	Gomutra	Kapha-vata śamanam, dīpanam	Cow urine enhances humoral, and cell-mediated immune response has bioenhancing property and antioxidant activity [23, 24]

10	Goat's milk	Vata-pitta śamanam	Hepatoprotective, lowers levels of liver damage by reducing ALT, AST, and gene expression CYP2E1 and TNF-*α* [25]

## Data Availability

All the data used in this case study are available as part of the article, and no additional source data are required.

## Supporting Information

Supporting Information Annexure 1: It includes the treatment charts of patients' with details on date, complaints, medications prescribed, and diet and lifestyle modifications advised.

Supporting Information Annexure 2: It includes the original lab data points used in Figures 2, 3, and 5.
