# Supplementary material for: A Case Report on Management of Liver Cirrhosis Using Ayurveda and Integrative Approach of Treatment
Source: Case Reports Hepatol. 2024 Nov 27;2024:1176751. doi: 10.1155/crhe/1176751 (PMC11617052; doi:10.1155/crhe/1176751)
Supplement: Supporting Information — Annexure 1: It includes the treatment charts of patients' with details on date, complaints, medications prescribed, and diet and lifestyle modifications advised. [file 1176751.f1.docx]

**Annexure 1**

**Supplementary Table 1: Treatment chart of Case 1**

| **Date** | **List of herbal formulations** | **Diet and lifestyle advised** |
| --- | --- | --- |
| 22-4-15 | 1. Vasaguluchyadi kashayam -3tsf-3tsf-3tsf before food 2. Arogyavardini Bati 2-0-2 before food 3. Nirocil tab 2-0-2 twice before food 4. Pranada tab 0-0-2 at bed time | 1. Buttermilk with food (rice/green gram) for one week 2. Bhoomiamalaki juice |
| 28-4-15 | 1. Triphala churna 2 tsf with 100ml gomutra, Vasaguluchyadi Kashayam -3tsf before food, 2. .Arogyavardini Bati 2 tab twice before food, 3. Nirocil tab 2 tab twice daily before food, 4. Pranada tab -2 tab at bed time | 1. Easily digestible foods like Pongal, Rice, Kichdi, thin dal, barley ganji, buttermilk |
| 5-5-2015 | 1. Triphala churna 2tsf with 100ml gomutra, 2. Vasaguluchyadi Kashayam -3tsf before food, Arogyavardini Bati 2 tab twice before food, 3. Nirocil tab 2 tab twice daily before food, 4. Pranada tab -2 tab at bed time | 1. Easily digestible foods like Pongal, Rice, Kichdi, thin dal, barley ganji, buttermilk |
| 14-5-15 | 1. Pippali ¼tsf with medicated  buttermilk 2. Vasaguluchyadi Kashayam -3tsf before food, 3. Arogyavardini Bati 2 tab twice before food, 4. Nirocil tab 2 tab twice daily before food | 1. Buttermilk prepared using  3tsf of dashamoola, shadanga, unarnava with 1 litre of milk and 1 litre of water, boil and reduced to 1 litre to make buttermilk. |
| 14-05-2015 to 06-06-2015 | 1. Vasaguluchyadi Kashayam -3tsf before food, 2. Arogyavardhini bati (0-2-2) 2 tab twice daily before food 3. Nirocil tab 2 tab twice daily before food Pippali ¼tsf with medicated  buttermilk 100ml of gomutra 100ml with 200ml of milk early morning at 7 am | 1. Medicated buttermilk prepared using  3tsf of dashamoola, shadanga, punarnava with 1 litre of milk and 1 litre of water, boil and reduced to 1 litre to make buttermilk. |
| 06/06/2015- Discharge medicines | 1. Bhumiamalaki swarasa 20-0-20 ml  2. Arogyavardhini rasa 1-1-1  3. Punarnava paneeya | 1. 5 tsf of Punarnava paneeya with 1.5 litres of water, boiled and reduced to 1 litre, filtered and taken frequently as drinking water. |
| 27-07-2015 | 1. Nityananda rasa 1 tab twice daily, 2. 0.5 tsf of triphala, Hinguwastaka, ajamoda with 10 tsf gomutra in the mornings, 3. Punarnava paneeya | 1. Ksheera patya 2. 5 tsf of Punarnava paneeya with 1.5 litres of water, boiled and reduced to 1 litre, filtered and taken frequently as drinking water. |
| 27-08-2015 to 18-09-2015 | 1. Nityananda rasa 1-0-1 twice daily before food 2. Triphala Churna 10tsfof gomutra , 1 tsf of triphala , ½ tsf of hingwastaka churna , ½ tsf ajamodadi churna 3. Punarnava 5 tsf , 1 ½ litre of water- boiled and reduced to 1 litre, filtered and used for frequent drinking 4. Chandraprabha vati 2-0-2 before food | 1. Abhyanga- ekanga with Dhanwantaram taila for 5 days followed by nadi sweda to the chest and back |
| 18-9-15 | 1. Nityananda rasa 1-0-1 twice daily before food 2. Triphala Churna 10 tsf of gomutra , 1 tsf of triphala , ½ tsf of hingwastaka churna , ½ tsf ajamodadi churna 3. Punarnava 5 tsf , 1 ½ litre of water- boiled and reduced to 1 litre, filtered and used for frequent drinking 4. Chandraprabha vati 2-0-2 before food | 1. Abhyanga- ekanga with Dhanwantaram taila for 5 days followed by nadi sweda to the chest and back |
| Advise on discharge | 1. Nityananda rasa 1-0-1 twice daily before food 2. Triphala Churna 10 tsf of gomutra , 1 tsf of triphala , ½ tsf of hingwastaka churna , ½ tsf ajamodadi churna 3. Punarnava 5 tsf mixed in 1 ½ litre of water, boiled and reduced to 1 litre, filtered and used for frequent drinking   Chandraprabha vati 2-0-2 before food | 1. Easily digestible foods like Pongal, Rice, Kichdi, thin dal, barley ganji, buttermilk |

* 1 tsf = 4.9289 mL

**Supplementary Table 2: Treatment chart of Case 2**

| **Date** | **Intervention (combination of)** | **Diet and lifestyle advised** |
| --- | --- | --- |
| 26-3-19 | 1. Punarnava gokshura paneeya, 2. 2tsf triphala with 200ml dasamoola paneeya, 3. Nirocil tab (0-3-3) after food 4. Vasaguluchyadi kashayam (3tsf-0-3tsf), 5. Gomutra hareetaki -2tsf with dasamoola paneeya at 6AM |  |
| 30-3-19 | 1. Punarnava gokshura paneeya, 2. 2tsf triphala with 200ml dasamoola paneeya, 3. Nirocil tab (0-3-3) after food 4. Vasaguluchyadi kashayam (3tsf-0-3tsf) | 1. Milk diet |
| 12-4-19 | 1. Punarnava gokshura paneeya, 2. 2tsf triphala with 200ml dasamoola paneeya, 3. Nirocil tab (0-3-3) after food 4. Vasaguluchyadi kashayam (3tsf-0-3tsf), 5. Ferbery syrup 2tsf thrice daily | 1. Red rice, jave godhi, green gram |
| 14-5-19 | 1. Punarnava gokshura paneeya, 2. Nirocil tab (0-3-3) after food 3. Vasaguluchyadi kashayam (3tsf-0-3tsf), 4. Ferbery syrup 2tsf thrice daily |  |
| 25-5-19 | 1. Punarnava gokshura paneeya, 2. 2tsf triphala with 200ml dasamoola paneeya, 3. Nirocil tab (0-3-3)after food 4. Vasaguluchyadi kashayam(3tsf-0-3tsf), 5. Gomutra hareetaki -2tsf with dasamoola paneeya 6am, 6. Ferbery syrup 2tsf thrice daily |  |
| 7-9-19 | 1. Punarnava gokshura paneeya, 2. 2tsf triphala with 200ml dasamoola paneeya, 3. Nirocil tab (0-3-3) after food 4. Dasamoola hareetaki 1tsf ,1tab mandoora vatakam twice daily | 1. Saindhava lavana 3/4tsf,sea salt 1/4tsf with buttermilk , sattu with javee godhi |
| 20-11-19 | 1. Punarnava gokshura paneeya, 2. 2tsf triphala with 200ml dasamoola paneeya, 3. Nirocil tab (0-3-3)after food 4. Dasamoola hareetaki 1tsf ,1tab mandoora vatakam twice daily | 1. tender papaya leaf juice 2tsf |
| 26-2-20 | 1. Punarnava gokshura paneeya, 2. 2tsf triphala with 200ml dasamoola paneeya, 3. Nirocil tab (0-3-3)after food 4. Dasamoola hareetaki 1tsf ,1tab mandoora vatakam twice daily, 5. Mahathiktaka kashayam (0-3tsf-3tsf) before food |  |
| 8-7-20 | 1. Nirocil tab (0-3-3)after food 2. Dasamoola hareetaki 1tsf ,1tab mandoora vatakam twice daily, 3. Mahathiktaka kashayam (0-3tsf-3tsf) before food |  |
| 16-1-21 | 1. All medications were on hold | 1. Papaya , guard varieties ,apple, kishmish ,chaas ,methi leaves, |
| 30-1-21 | 1. Punarnava gokshura paneeya, 2. 2tsf triphala with 200ml dasamoola paneeya, 3. Nirocil tab (0-3-3)after food 4. Dasamoola hareetaki 1tsf ,1tab mandoora vatakam twice daily, 5. Mahathiktaka kashayam (0-3tsf -3tsf) before food |  |
| 27-2-21 | 1. punarnavagokshuradasamoola, 2. Mahathiktaka kashayam (0-3tsf -3tsf) before food, 3. Avipathi churna with triphala |  |
| 30-4-21 | 1. Punarnava gokshura dasamoola, 2. Mahathiktaka kashayam (0-3tsf -3tsf) before food, 3. Avipathi churna with triphala | Continue milk and buttermilk diet |

* 1 tsf = 4.9289 mL

**Supplementary Table 3: Treatment chart of Case 3**

| **Date** | **List of herbal formulations** | **Diet and lifestyle advised** |
| --- | --- | --- |
| 14-09-2017 | 1. Bhumyalaki swarasa 2tsf , guduchi swarasa 2tsf to be taken at 7pm 2. Avipathi choornam 15gms to be taken at 7am with warm water followed by buttermilk diet till 12.00pm, 3. Tab Liv 52 to be taken thrice daily 20 min after food with warm water 4. Punarnava paneeyam | Pattabandhana for 9 days |
| 23-09-2017 Advise on discharge | 1. Bhumiamalaki swarasa 2tsf , guduchi swarasa 2tsf at 7pm  2. avipatti choornam ½ tsf with warm water at 7am  3. 1/2tsf ajamodadhi choornam with buttermilk at 12PM   1. Punarnava paneeya -3tsf Punarnava choornam boiled in 2 litres of water and reduced to 1.5 litre | 1. Pattabandhana at home |
| 17-10-2017 | 1. Bhumiamalaki swarasa 2tsf , vasa swarasa 2tsf  twice daily, 2. Punarnava paneeyam | 1. Milk diet |
| 12-05-2017 | 1. Avipatti churnam 1/2-0-0 2. 1/2 tsf with butter milk , 1/2 tsf of ajamoda choorna, 3. Punarnava paneeyam, shadanga paneeyam 4. Nirocil Tab 2-2-2 before food, 5. Vayu Gulika 2-2-2 between each meal, 6. Himcocid Syr sos before food, 7. Kottamchukkadi Tailam for external application |  |
| 21-04-2018 | 1. Punarnavadi kashayam 4tsf-0-4tsf , 8tsf warm water at 6 am - 6 pm twice daily, 2. Hingutriguna Thailam 3tsf-0-0 once daily at 6 am with Punarnavadi Kashayam , 3. Drakshadi Kashayam 4tsf-0-4tsf , 8tsf warm water at 11am and 10pm , 2 tsf honey, Panchakola Choornam 30gms , 1/2 litre milk , 1.5 litre water -boiled and reduced to 1/2 litre make into curd and then into buttermilk used for eating, 4. Lohasavam 2tsf,punarnavasava 2tsf , 4tsf warm water after lunch, | 1. Continue Buttermilk Diet, Continue 2 eggs whites per twice daily, Moong dal soup, Milk every day. |
| 19-05-2018 | 1. Panchakola Choornam 30gms , 1/2 litre milk , 1.5 litre water -boiled and reduced to 1/2 litre and made into buttermilk used for drinking, 2. Punarnavadi kashayam , vasaguluchyadi kashyam 0-4tsf-4tsf before food with 8tsfof warm water, 3. Hinguthariguna Thailam 3tsf-0-0 once daily at 6 am with Punarnavadi kashayam ,vasaguluchyadi kashayam, 4. Hinguvachadi Gulika 0-2-2 ,2 at lunch and dinner with kashayam | 1. 2 egg whites daily Mudga Yusha Panchakola Yavagu, Buttermilk Diet. |
| 21-05-2018 | 1. Vasaguduchyadi Kashayam 0-8tsf-8tsf twice daily before meals at 6am- 6 pm, 2. Punarnavadi Kashayam 0-8tsf-8tsf twice daily before meals, 3. Hingutriguna Taila 4tsf-0-0 at 6am with 100ml warm milk for Nitya Virechana, 4. Hinguvachadi Gulika 0-2-2 twice daily before meals with warm water, 5. Vasa Svarasa 3tsf-0-3tsf after food with Honey , 6. Bhumiamalaki Svarasa 4tsf -0-4tsf before food with Honey, 7. Pippali Rasayana with Goats milk for 20 days |  |
| 16-07-2018 Advise on discharge | 1. 1.Hingutriguna tailam 3tsf-0-0 6am with 100ml warm milk. 2. Vasaguduchyadi Kashayam 0-4tsf-4tsf twice daily with 8tsf warm water before meals 3. Punarnavadi Kashayam 0-4tsf-4tsf twice daily before meals,with 8tsf warm water before meals 4. Indukanta ghritha 0-0-2tsf at 6:30pm with above kashayam 5. Tab.Hinguvachadi Gulika 0-1-1 with swarm water before meals 6. Vasa swarasa 0-2tsf-2tsf at 11am and 4pm with honey 7. Bhumiamalaki swarasa 0-2tsf-2tsfat 11am and 4pm | 1.Pattabandhana at home |
| 01-08-2018 | 1. Panchakola Choornam 30gms , 1/2 litre milk , 1.5 litre water -boiled and reduced to 1/2 litre make into curd and made into buttermilk used for eating, 2. Hinguthariguna Thailam 3tsf-0-0 once daily at 6 am with 6tsf gomutra, 3. Punarnavadi kashayam,Indukantham kashayam 0-4tsf-4tsf  before food with 8tsf of warm water, 4. Hinguvachadi Gulika 0-1-1 with kashaya, 5. Liv 52 DS 0-0-2 ,1 hour before food, |  |
| 04-01-2019 | 1. Mandoora Vatakam 1-0-0 with diet, 2. Dasamoola Rasayana 2tsf-0-0 empty stomach 3. Bhumiamalaki swarasa 4tsf twice daily 1 hour before meals, with buttermilk | 1. Evening-Pomegranate juice - 60 ml Morning- Raisins,12 Figs,2 Dates Soaked overnight take in early morning once. 2. Morning Diet -Kharjura , Dry raisins , Anjir , - Mantha to be prepared 1 day before and consumed the next day Evening - 1 pomegranate. |

* 1 tsf = 4.9289 mL
