# Supplementary material for: A Case Report on Management of Liver Cirrhosis Using Ayurveda and Integrative Approach of Treatment
Source: Case Reports Hepatol. 2024 Nov 27;2024:1176751. doi: 10.1155/crhe/1176751 (PMC11617052; doi:10.1155/crhe/1176751)
Supplement: Supporting Information — Annexure 2: It includes the original lab data points used in Figures 2, 3, and 5. [file 1176751.f2.docx]

**Annexure 2: Laboratory data**

**Case 1:**

|  | **Baseline** | **60 days** | **120 days** | **180 days** |
| --- | --- | --- | --- | --- |
| **Total Bilirubin mg/dl** | 3.31 | 2.1 | 0.9 | 0.7 |
| **Direct Bilirubin mg/dl** | 2.1 | 1.4 | 0.4 | 0.3 |
| **SGOT u/l** | 304 | 20 | 18 | 20 |
| **SGPT u/l** | 200 | 28 | 28 | 22 |
| **Trans Iliac Diameter cms** | 90.5 | 85 | 105 | 108 |
| **Fluid thrill** | Positive |  | positive | positive |
| **Shifting Dullness** | Noted | absent |  | noted |
| **Transpyloric Diameter cms** | 80 |  |  |  |
| **Trans-Umbilicus Diameter cms** | 90 |  |  |  |

**Case 2:**

|  | **Baseline** | **60 days** | **240 days** | **330 days** | **660 days** | **750 days** |
| --- | --- | --- | --- | --- | --- | --- |
| **Total Bilirubin mg/dl** | 1.48 | 1.6 | 1.7 | 1.6 | 1.71 | 1.8 |
| **Direct Bilirubin mg/dl** | 0.49 | 0.5 | 0.42 | 0.47 | 0.47 | 0.4 |
| **SGOT u/l** | 35 | 20 | 17 | 21 | 23 | 40.5 |
| **SGPT u/l** | 76 | 39 | 45 | 48 | 49 | 97.4 |

**Case 3:**

|  | **Baseline** | **60 days** | **120 days** | **180 days** | **240 days** | **300 days** | **360 days** | **420 days** | **720 days** |
| --- | --- | --- | --- | --- | --- | --- | --- | --- | --- |
| **Total Bilirubin mg/dl** | 2.6 | 2.8 | 2.3 | 3.1 | 2.6 | 1 | 1.3 | 2.2 | 1.5 |
| **Direct Bilirubin mg/dl** | 1.5 | 1.9 | 1 | 1.7 | 1.2 | 0.4 | 0.5 | 1.1 | 0.6 |
| **SGOT u/l** | 54 | 45 | 45 | 39 | 36 | 62 | 47 | 47 | 26 |
| **SGPT u/l** | 20 | 17 | 15 | 19 | 18 | 27 | 28 | 18 | 22 |
